# Selection and enrichment of microbial species with an increased lignocellulolytic phenotype from a native soil microbiome by activity-based probing

**DOI:** 10.1038/s43705-023-00305-w

**Published:** 2023-09-30

**Authors:** Nicholas J. Reichart, Andrea K. Steiger, Elise M. Van Fossen, Ryan McClure, Herman S. Overkleeft, Aaron T. Wright

**Affiliations:** 1 Biological Sciences Division, Pacific Northwest National Laboratory, Richland, WA USA; 2 Leiden Institute of Chemistry, Leiden University, Leiden, The Netherlands; 3 Department of Biology, Baylor University, Waco, TX USA; 4 Department of Chemistry and Biochemistry, Baylor University, Waco, TX USA

**Keywords:** Microbiome, Soil microbiology

## Abstract

Multi-omic analyses can provide information on the potential for activity within a microbial community but often lack specificity to link functions to cell, primarily offer potential for function or rely on annotated databases. Functional assays are necessary for understanding in situ microbial activity to better describe and improve microbiome biology. Targeting enzyme activity through activity-based protein profiling enhances the accuracy of functional studies. Here, we introduce a pipeline of coupling activity-based probing with fluorescence-activated cell sorting, culturing, and downstream activity assays to isolate and examine viable populations of cells expressing a function of interest. We applied our approach to a soil microbiome using two activity-based probes to enrich for communities with elevated activity for lignocellulose-degradation phenotypes as determined by four fluorogenic kinetic assays. Our approach efficiently separated and identified microbial members with heightened activity for glycosyl hydrolases, and by expanding this workflow to various probes for other function, this process can be applied to unique phenotype targets of interest.

## Introduction

The genomes of microorganisms within soil, animal hosts, and aquatic systems encode for an extensive functional capacity involving myriad biochemical activities. These include biogeochemical cycling, synthesis of signaling molecules and vitamins, mineralization, metabolism of complex molecules, detoxification, and other activities [1, 2]. Understanding these microbial functions and their environment-specific responses is often restricted by approaches that rely on functional inference from metagenomes [3] or indirect measurements. Improving identification and understanding of the community members responsible for specific functions will enable enhanced knowledge of host-associated and ecological roles of microbiomes, improve predictions of microbiome change due to perturbations, and create opportunities for selectively harnessing and applying microbes for synthetic biology or bioengineering [4].

The networks of microbial species and the diversity of their genomes means that certain species will only express sets of functions that drive their own fitness, either through synergistic or antagonistic interspecies interactions [5]. This causes a disconnect between the genomic potential of a species (all processes it *could* express based on sequenced genomes) and the phenotype of a species (all processes it *does* express as functional enzymes) during growth in a community [6]. Because of this disconnect, genomic and metagenomic analyses of microbiomes are insufficient to gain detailed knowledge of the processes and functions these communities perform. Proteomics or transcriptomics can determine species expressing proteins or transcripts relevant to a function, but the abundance-based measurements do not account for regulation, such as inactivated enzyme precursors as zymogen forms, inhibited proteins, or post-transcriptional/translational modifications. Additionally, metagenomes used for microbiome proteomics/transcriptomics are often poorly functionally annotated, resulting in many proteins or transcripts and the species expressing them remaining unidentified [7, 8]. Metabolomic analysis detects molecules exuded by microbial communities, but these can be difficult to cross-reference to a specific function and are not species-specific [9].

Activity-based protein profiling (ABPP) for functional annotation of microbial communities can better address the disconnect of genomics to phenotype [10, 11]. ABPP uses activity-based probes (ABPs), which are small-molecule substrates composed of a reactive warhead group that selectively and covalently binds to active enzymes, and a reporter tag attached through a linker that permits characterization through fluorescence visualization of the bound, active enzyme [12–15]. ABPs have strong specificity when attaching to enzymes performing a function of interest. This is an advantage over other methods which may only work as a proxy for activity [16] or can suffer from metabolic cross-feeding [17]. Additionally, the nondestructive and nontoxic nature of many ABPs enables downstream characterization of viable microbes following probe labeling. Recently, we developed an ABPP approach to select and characterize microbial species expressing active enzymes related to a specific phenotype. Live cells from gut microbiome samples were labeled with an ABP-targeting β-glucuronidase prior to taxonomic characterization following fluorescence-activated cell sorting (FACS) of fixed cells [18, 19]. This ABPP-derived approach works with samples from complex environments, enables characterization of non-isolated microbes, and provides a high throughput, function-forward analysis of complex microbial communities.

From a synthetic biology or bioengineering perspective, ABPP has the potential to derive improved microbes and consortia for the breakdown of lignocellulose through valorization reactions. Valorization of lignocellulosic biomass by microbial organisms to degrade cellulose and hemicellulose as cellobiose and xylose, particularly from soil, has generated significant interest and fueled multiple bioengineering endeavors. To date, ABPP has played a role in characterizing lignocellulose degradation through ABPs [20] for enzymes such as exogluconases [21] and exoxylonases [22]. Herein, we describe an ABPP approach that enables function-selective isolation of *living* microbes (ABP-FACS, a significant step forward from the previous probe and lignocellulose work that has been published) paired with kinetic activity analysis and taxonomic sequencing of the sorted, functional consortia to link quantifiable substrate consumption to specific shifts in the composition of a complex, native soil microbial community. The application of ABPs to lignocellulose functions of the soil microbiome has the potential to both expand our fundamental knowledge of this phenotype as well as lead to new potential for biotechnological application of microbial systems. This approach can be extrapolated to various other functions (e.g., plastic degradation, carbon sequestration) and ecosystems (e.g., wastewater, ocean, gut microbiome). The application of our approach to these communities has the potential to isolate populations of microbes that may be well-suited to carrying out economically viable functions, to illuminate which members of a community are performing certain functions, and to demonstrate how these phenotype profiles may shift as a function of environmental or interspecies interaction cues.

## Methods

### Soil incubation and cell extraction

Approximately 3 kg of unmanaged marginal soil (pH 8) was collected from the Pacific Northwest National Laboratory field site in Prosser, Washington (46° 15′ 04″ N and 119° 43′ 43″ W) [23], homogenized, sieved (4 mm mesh size), and stored at 4 °C until further processing. Probe specific incubation experiments were prepared with four replicate incubations of 2 g of soil in 50 mL tubes. The soil was wetted with 200 µL of a water solution containing either 2% cellobiose or xylan for a 0.2% final substrate concentration. The tubes were incubated covered with a Breathe-Easy membrane (Diversified Biotech, Dedham, Massachusetts) at 25 °C with 1 L of water in a beaker to generate humidity in the incubator. Cellobiose experiments were incubated for three days and xylan experiments for nine days [22].

Following the incubations, a Nycodenz (Serumwerks Bernburg AG, Bernburg, Germany) density gradient cell extraction was performed. For each incubation tube containing 2 grams of soil, 4 mL of 1× tris-buffered saline (TBS) was added and the tube was vortexed for 15 min. The samples were allowed to gravity settle for 5 min prior to moving the supernatant to a new tube for subsequent Nycodenz gradients. To the remaining soil, an additional 2 mL of 1× TBS was added and the steps repeated combining both collections of gravity settled supernatant. To create a gradient for extraction, 5 mL of 40% Nycodenz was gently layered underneath the collected supernatant followed by an additional 2 mL of 80% Nycodenz added below the 40% layer. The tubes containing the collected supernatant samples and Nycodenz layers were centrifuged for 15 min at 5000 rcf with slow speed ramp and no brake. After centrifugation, the top and middle layers containing the cells were aspirated and transferred to a new 15 mL tube. Equal volume amounts of 1× TBS were added to the cell solutions. After a brief vortex, the tubes were centrifuged for 15 min at 7000 rcf to pellet the cells. The supernatant containing the remaining Nycodenz was discarded, and the pellet was suspended in 1 mL of 1× TBS and transferred to a 1.5 mL tube. From each extracted sample, including a blank extraction from the reagents used, 100 µL was removed for later 16S rRNA gene amplicon sequencing. The remaining 900 µL in the tube was then centrifuged for 7 min at 9000 rcf, supernatant discarded, and pellet suspended in 1 mL 3-(N-morpholino) propanesulfonic acid (MOPS media; Teknova, Hollister, California) containing the corresponding carbon source (0.2% cellobiose or xylose). Samples were incubated overnight at 25 °C shaking at 350 RPM. The next day, an additional 100 µL sample was collected for 16S rRNA gene processing.

### Activity-based probe labeling paired to live cell fluorescence-activated cell sorting

After overnight growth, the extracted cell samples from four replicates were combined into one tube per substrate. Multiple replicates were maintained until this step to mitigate the variability in microbial growth patterns and to maximize the number of cells recovered for subsequent processing. The combined sample was centrifuged for 7 min at 9,000 rcf before the pellet was suspended in 1 mL MOPS buffer without a carbon source. The sample was then split into three tubes, 500 µL for probe labeling and staining with a Syto nucleic acid stain (Invitrogen, Waltham, Massachusetts), 250 µL for only staining with Syto, and 250 µL for a no-fluorescence control. For the cellobiose incubated sample, the GH4a probe [21] was used to target glucosidase and cellobiosidase activity, and for the xylan incubated samples, the SYF161 probe [22] was used to target xylosidase and xylobiosidase activity. The GH4a probe was conjugated to a BODIPY (excitation = 502 nm, emission = 511 nm) tag requiring Syto59 (Invitrogen; excitation = 622 nm, emission = 645 nm) to be used as the general nucleic acid stain, while SYF161 was conjugated to a Cy5 (excitation = 651 nm, emission = 670 nm) tag and coupled with Syto9 (Invitrogen; excitation = 485 nm, emission = 498 nm). The probes were dissolved in DMSO and the final concentration of 100 µM and 10 µM were used for GH4a and SYF161, respectively. To the “Syto only” and “no-fluorescence” control samples, equivalent volumes of DMSO were added. Probe labeling of the extracted cell populations occurred for one hour at 25 °C with shaking at 350 RPM. Cells were washed once with MOPS buffer by centrifugation for 7 min at 9000 rcf to remove unbound probe before final suspension in twice the volume used for labeling. To the Syto containing samples, a final concentration of 5 µM was used for either Syto59 or Syto9 and were added to the samples after probe labeling but before being loaded onto the cell sorter. Prior to loading any sample onto the cell sorter, samples were filtered through a 35-µm mesh filter cap.

The Sony SH800 cell sorter was used for all sorting experiments with a four-laser set up (405, 488, 561, and 638 nm) and a 100-um sorting chip. The samples were sorted using the “Normal” sort mode into two-way 1.5 mL tubes using a forward scatter (FSC) threshold value of 0.05%. The gating strategies for both probe types are depicted in Supplementary Figs. 1 and 2 (GH4a- and SYF161-ABP, respectively). Initial gates were established on forward scatter—area by back scatter—area (FSC-A x BSC-A) and then forward scatter—height by forward scatter—width (FSC-H and FSC-W) to select for cells of expected size. The third gate was established with the Syto only control and no-fluorescence control to select for nucleic acid containing event droplets indicative of microbial cells in the sorted droplet and not soil debris or instrument noise. Cells collected from this gate are referred as “Bulk” samples. Once the Bulk sample gate was determined, corresponding Syto stain was added to the probe-labeled samples for a 5 µM final concentration. Using all three sample types, the fourth gate was established to collect for probe positive events, referred to as “Enriched” samples, and probe negative events, referred to as “Depleted” samples. Each subsequent gate type was nested within the previous to work as a step-wise process for selection of ABP-labeled cell events.

Sorting for Bulk, Depleted, and Enriched samples was done iteratively, collecting 50,000 events for later kinetic assay incubations (kinetic samples) or collection of 100,000 events for direct 16S rRNA gene amplicon processing [16]. Kinetic assay sorted samples were collected as four replicates, while five replicates were collected for direct 16S rRNA gene sequencing. Throughout each day of sorting (GH4a and SYF161 sorting occurred on separate days), three samples were collected from the sheath fluid waste stream to track potential contamination or any leftover microbes in the fluidics lines.

### Kinetic assays

**Fig. 1:**
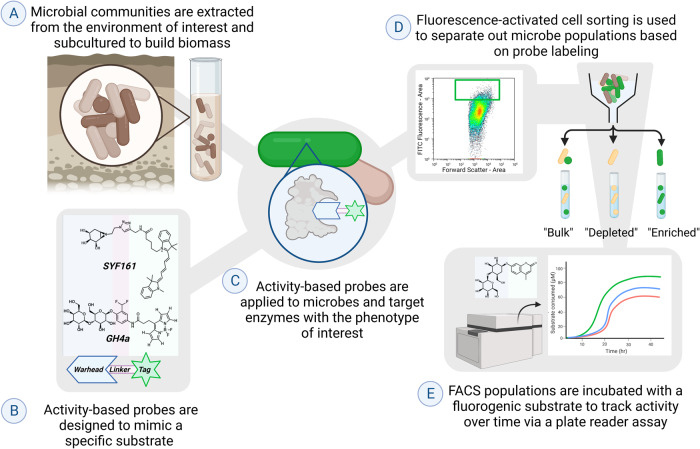
Workflow diagram for the application of ABP-FACS for kinetic MUB assay. **A** Microbes are extracted from an environmental soil sample and incubated in media overnight. **B** Activity-based probes are developed with a warhead to target specific enzymes, a linker as a bridge to the functional tag, and a fluorophore tag to allow for downstream analyses. **C** The designed activity-based probes are applied to the cell community to target enzymatic reactions for the phenotype of interest. **D** Fluorescence-activated cell sorting is used to sort populations of cells depending on the presence or absence of probe labeling. **E** Sorted microbial populations are plated and incubated with fluorogenic compounds to track enzymatic activity over time as a proxy for phenotypic activity. Image generated using BioRender.

After the completion of sorting, the kinetic samples were transferred into 10 mL culture tubes containing 4 mL of MOPS media containing either 0.2% cellobiose or xylose. To detect kinetic activity, fluorogenic substrates composed of methylumbelliferone (MUB) conjugated to substrate analogs (referred to hereafter as “MUBstrates”) were added to the cultures [24] at a final concentration of 100 µM. For samples probed with GH4a, either a glucose-MUB or cellobiose-MUB (Sigma-Aldrich, St. Louis, Missouri) was used. For samples probed with SYF161, either a xylose-MUB or xylobiose-MUB (Sigma-Aldrich) was used. The full workflow of our approach from soil incubation through probe labeling, FACS, and kinetic assay is depicted in Fig. 1.

### Plate reader evaluation of continuous kinetic assays

From the 4 mL cultures containing sorted cells and MUBstrates, an initial 40 µL sample (estimated to have 500 cells) was collected in triplicate and transferred to a 384-well black walled clear bottom plate for a continuous kinetic reading. The plate was incubated in a Synergy H1 plate reader (BioTek, Winooski, Vermont) at 25 °C with shaking (250 RPM). Cellular growth and activity were monitored by collecting the optical density (OD, 600 nm) and fluorescence from hydrolyzed MUBstrate (330/450 nm), respectively, every 15 min as the continuous kinetic assay. The assay was continued for 48 and 100 h for the GH4a and SYF161 probes, respectively.

### Culture-tube incubations

The 4 mL cultures were incubated at 25 °C with shaking (250 RPM) and cellular growth and kinetic activity were monitored by collecting the optical density (OD, 600 nm) and fluorescence from hydrolyzed MUBstrate (330/450 nm), respectively, on a Synergy H1 plate reader (BioTek) as the discrete kinetic assay. For this, triplicate 40 µL technical replicates were collected from the four biological replicate culture tubes for each MUBstrate at four time points over the course of the incubation for growth and activity analysis and later 16S rRNA gene analysis. Time points were selected based on previous experiments. GH4a probed samples were interrogated 15, 25, 35, and 45 h into the incubations, whereas SYF161 probed samples were collected 28, 38, 48, and 64 h into the incubation. Following the plate reader screening, the discrete time point samples were collected from the plate and processed for 16S rRNA gene sequencing. We sampled for 16S rRNA gene sequencing from the culture tubes to allow the previously described continuous kinetic assays to run without destructive sampling at the determined time points. We used the entire 40 µL replicates to ensure enough biomass was used for DNA input for PCR.

### Amplicon sequencing

All samples throughout the experiment labeled for 16S rRNA gene sequencing at the time of sampling were centrifuged for 7 min at 9000 rcf, supernatant discarded, and pellet suspended in 20 µL of nuclease-free water. These samples were frozen until later processing. Samples collected and frozen for 16S rRNA gene amplicon sequencing were thawed and transferred to 96-well plates. Plates were subjected to three rounds of freeze thaw; 20 min at −80 °C followed by 10 min at 99 °C. Following the last round, 10 µL of each lysed cell sample was transferred to a new plate containing necessary barcoded forward primer and reagent cocktail mixture. The forward primer (1 µL, 0.2 µM final concentration) targeted the 515 V4 region (5′-GTGYCAGCMGCCGCGGTAA-3′) [25] and contained unique 12 base barcodes. The reagent cocktail mixture contained 20 µL Platinum II Master Mix (Invitrogen; 0.8× final concentration), 1 µL 806 reverse primer (5′-GGACTACNVGGGTWTCTAAT-3′; 0.2 µM final concentration) [26], and 18 µL water to make a 50 µL final volume reaction. The plates were sealed and loaded onto a ProFlex PCR Systems thermocycler (Applied Biosystems, Waltham, Massachusetts) and run under the following conditions: initial hot start at 74 °C for 3 min, followed by 35 cycles of 94 °C for 60 s, 55 °C for 45 s, and 74 °C for 90 s, before a final extension step at 74 °C for 10 min, as recommended from the Earth Microbiome Protocol. After completion of PCR, samples were assayed for DNA concentration using Quant-iT PicoGreen dsDNA assay kit (Invitrogen) in triplicate reactions. A total of 200 ng per sample was pooled into a single tube, or up to 40 µL if 200 ng could not be achieved, and cleaned of excess primers using a Zymo Clean and Concentrator – 100 kit (ZymoResearch, Irvin, California).

The pooled and cleaned sample was further diluted to 2 nM and the recommended protocol for Illumina v2 500 cycle chemistry for the MiSeq was followed. PhiX was used at a final 15% concentration and mixed into the sample prior to loading on the MiSeq. Samples relating to cellobiose incubations were pooled and sequenced on a separate flow cell from the xylose incubation samples. Data was exported from the MiSeq as demultiplexed fastq files.

### Data analysis

Demultiplexed reads were processed using Qiime2 (version 2021.4) [27]. The adapter sequences were trimmed, and reads were truncated to maintain quality using DADA2 [28] (forward: 180 bp, reverse: 140 bp). Further within DADA2 processing using the default parameters, the reads were denoised, merged and chimera-checked resulting in 19,490,457 reads for cellobiose and xylose samples combined. Taxonomy was assigned as amplicon sequence variants (ASVs) using the Silva SSU database release 138 with the classify-sklearn plugin within the Qiime2 environment. Further read processing occurred using R [29]. The package decontam [30] was run to remove ASVs identified as contaminants using a 0.5 threshold with the Prevalence model. Control samples were collected from extraction and processing reagents, sheath fluid and FACS lines, and PCR negatives to track potential contamination. Samples containing less than 5000 reads were discarded, as were singleton ASVs. The remaining reads were analyzed using Phyloseq [31], vegan [32], and DESeq2 [33] packages for diversity metrics and statistical tests with ggplot for figure generation.

## Results

### Phenotype selection in a soil microbiome results in activity-dependent isolation of glucose, cellobiose, xylose, and xylobiose degraders

Our experimental pipeline connected desired phenotypes to community characterization by coupling kinetic data to 16S rRNA gene sequencing via activity-based probes (ABPs). Soil extracted microbial communities were labeled with ABPs targeting either cellulase or xylanase activity. After labeling, live microbes from these communities were sorted based on the fluorescent signal from the conjugated ABP into three subpopulations for each ABP used: “enriched” were members containing the ABP-associated fluorescence, “depleted” were members that did not possess the ABP-associated fluorescence and “bulk” was comprised of the overall microbial population as determined by nucleic acid stain. During the post-sort incubation for kinetic activity, the OD values of samples were recorded to assay viability (Supplementary Fig. 3).

To detect kinetic activity, each subpopulation was then cultured in media containing fluorogenic substrates composed of methylumbelliferone (MUB) conjugated to substrate analogs (“MUBstrates”) [24]. A key component of this timeline was the ability to identify active community members by connecting kinetic assays to 16S rRNA gene sequencing. This was accomplished by setting up cultures for 16S rRNA gene analysis alongside the kinetic assay plates, from which samples were taken at various time points, adding a temporal dimension to the 16S rRNA gene analysis.

**Fig. 2:**
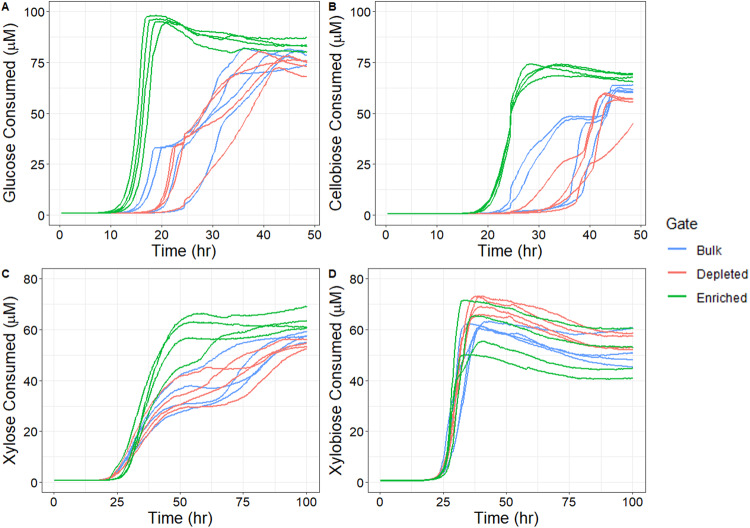
Kinetic assay of four biological replicates showing the averages of three technical replicates for each line. Values shown for time in hours of assay and substrate consumed as measured by fluorescence intensity converted to MUB concentration in µM. **A** GH4a sorted populations with glucose-MUB. **B** GH4a sorted populations with cellobiose-MUB. **C** SYF161 sorted populations with xylose-MUB. **D** SYF161 sorted populations with xylobiose-MUB.

Following soil extraction, probe labeling and FACS, the cellulase or xylanase activities of the probe-enriched microbial subpopulations were compared via kinetic MUBstrate assays. For all but one of the activities investigated, ABP enrichment produced a microbial community with increased functional activity for the desired phenotype (Fig. 2). The difference in activity for enriched populations versus bulk or depleted populations was most pronounced between 15 and 35 h for glucose-MUB and 20 to 40 h for cellobiose-MUB. Enriched populations for glucose-MUB reached peak MUBstrate consumption at 20.75 h consuming an equivalent of 95.5 µM (Fig. 2A). As each sample was provided with 100 µM MUBstrate, it is likely that the GH4a-enriched sample consumed all the glucose-MUB that was available in the assay. At the same time point, bulk and depleted populations had consumed only 18.7 and 8.4 µM, translating to a five and ten-fold difference in activity respectively between the enriched and non-enriched populations. This trend was consistent for cellobiose-MUB with maximum MUBstrate consumption of 71.9 µM for the enriched population at 33.5 h compared to bulk with only 23.9 µM consumed and depleted with 9.4 µM consumed (Fig. 2B).

Xylose-MUB showed moderate differences during the incubation whereas xylobiose-MUB had no discernable differences for enriched populations compared to bulk or depleted populations. Differences between populations for xylose-MUB appeared most pronounced around 50 h of incubation, but all populations continued to increase in MUBstrate consumption to the end of the incubation at 100 h where the enriched population recorded the highest level of MUBstrate consumed at 63.5 µM compared to bulk with 57.2 µM and depleted with 54.0 µM consumed (Fig. 2C). All populations incubated with xylobiose-MUB reached their maximum MUB concentration around 35–40 h (Fig. 2D) with a maximum MUBstrate consumption of 60.2 µM at 38.75 h for enriched populations. Bulk and depleted were within a similar range to enriched populations at 38.75 h having consumed 60.5 and 70.1 µM, respectively. We applied two ABPs to a soil microbiome with four MUBstrate kinetic assays to screen for phenotypic enrichment of lignocellulose-degrading functions. Through our approach, three of the four populations had at least moderate differences of the ABP-enriched communities showing the utility of our pipeline and the potential phenotypic heterogeneity present in a soil microbiome.

Culture tubes that were used for discrete kinetic readings and subsampling the microbial community through 16S rRNA sequencing generated different trends, however, glucose-MUB and cellobiose-MUB had the enriched populations reaching maximum MUBstrate consumption first out of the three populations (Supplementary Fig. 4A, B). More variability was seen in xylose-MUB incubation with each populations reaching max consumption at the final sampling time point of 64 h. Xylobiose-MUB max consumption occurred at the third time point after 48 h and did not increase in total MUBstrate consumption through the final sampling time point at the 64 h (Supplementary Fig. 4C, D). These kinetic data are evidence that ABPs reliably target and tag microbes possessing the function of interest for FACS for glucose and cellobiose consuming phenotypes.

### Differences in richness and evenness for ABP-enriched samples for all MUBstrates

**Fig. 3:**
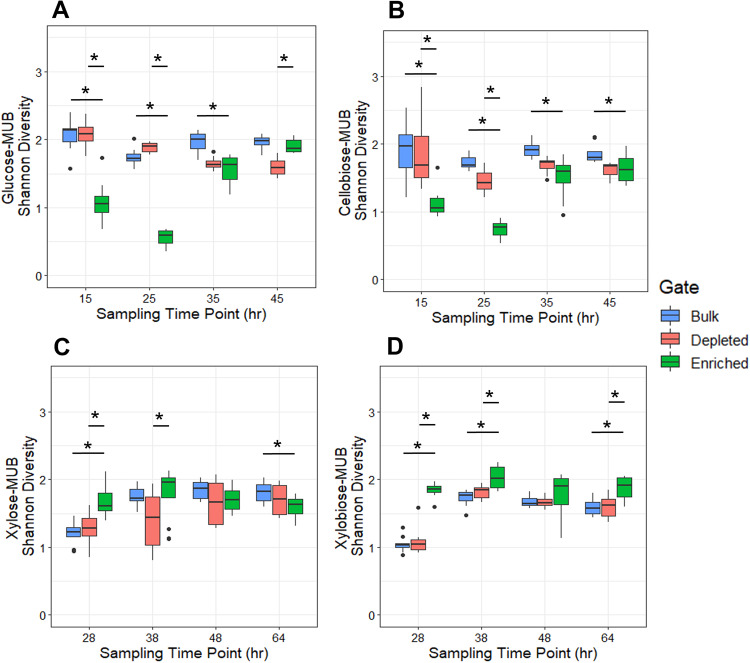
Shannon Diversity of probe sorted populations at four time during the kinetics incubation. **A** GH4a sorted populations incubated with glucose-MUB with time points occurring at 15, 25, 35, and 45 h. **B** GH4a sorted populations incubated with cellobiose-MUB with time points occurring at 15, 25, 35, and 45 h. **C** SYF161 sorted populations incubated with xylose-MUB with time points occurring at 28, 38, 48, and 64 h. **D** SYF161 sorted populations incubated with xylobiose-MUB with time points occurring at 28, 38, 48, and 64 h. Pairwise Wilcoxon test performed to determine significance for bulk or depleted Shannon Diversity compared to enriched samples. Significance (*p* < 0.05) is denoted with a star (*).

To further describe the bulk, depleted, and enriched populations, we performed 16S rRNA gene amplicon sequencing in a temporal fashion during the discrete kinetic assays. After each sampling time point, the samples were processed for sequencing. We detected 2409 amplicons sequence variants (ASVs) across 651 samples composed of 19,490,457 merged reads. The average Shannon diversity for the cellobiosidase targeting GH4a probe-enriched samples increased after the 35- and 45-h sampling compared to the earlier 15- and 25-h time points (Fig. 3A, B). Conversely, GH4a bulk and depleted samples were more consistent across the time points. Pairwise Wilcoxon tests were conducted at each time point to compare the Shannon diversity of bulk, depleted, and enriched samples, with the different MUBstrates separately. The Shannon diversity of enriched samples compared to bulk samples were significantly less at the first three time points (*p* < 0.5), whereas differences for enriched samples compared to depleted samples were significantly lower at 15 and 25 h, and higher at 45 h for the glucose-MUB. Additionally, the cellobiose-MUB had significant differences at all four time points for enriched compared to bulk, and only the first two time points for enriched compared to depleted with the enriched samples being lower in diversity across these time points.

Shannon diversity for xylose- and xylobiose-MUB samples sorted from the xylobiosidase targeting SYF161 probe remained consistent for enriched samples, with bulk and depleted samples consistent for the later time points after a low Shannon diversity at 28 h for time point 1 (Fig. 3C, D). Xylose-MUB Shannon diversity was significantly higher for enriched compared to bulk after the 28-h time point and lower at the conclusion of incubation at 64 h. Enriched compared to depleted was significantly higher for time points 1 and 2 at 28 and 38 h, respectively, for the xylose-MUB. For both enriched compared to bulk and enriched compared to depleted for xylobiose-MUB, significance was found after 28, 38, and 64 h but not at the 48-h time point, with the enriched samples consistently higher in Shannon diversity. All statistical values are reported in detail in Table 1. Overall, the temporal sampling for α-diversity during the MUBstrate assays showed high variation in the richness and evenness of the ABP-applied samples throughout the course of incubation.

**Table 1:** Shannon Diversity pairwise comparisons for ABP-FACS incubated populations.

Time point	Sample comparison	Glucose	Cellobiose	Xylose	Xylobiose
TP1	Bulk v Depleted	0.97	0.73961	0.44283	0.84
	Bulk v Enriched	**0.000065**	**0.0002**	**0.0000044**	**0.0000011**
	Enriched v Depleted	**0.000069**	**0.00066**	**0.00011**	**0.0000011**
TP2	Bulk v Depleted	**0.0018**	**0.00027**	0.0684	0.078
	Bulk v Enriched	**0.0000011**	**0.0000011**	0.0684	**0.0000089**
	Enriched v Depleted	**0.0000011**	**0.0000011**	**0.0087**	**0.0027**
TP3	Bulk v Depleted	**0.000033**	**0.000016**	0.57	0.98
	Bulk v Enriched	**0.000033**	**0.00011**	0.13	0.13
	Enriched v Depleted	0.59	0.19781	0.76	0.13
TP4	Bulk v Depleted	**0.0000044**	**0.0000044**	0.291	0.8874
	Bulk v Enriched	0.27	**0.038**	**0.027**	**0.0043**
	Enriched v Depleted	**0.0000044**	0.717	0.291	**0.0068**

Pairwise comparisons of the Shannon Diversity using Wilcoxon rank sum exact test with Benjamini–Hochberg adjusted *p*-values. Values in bold represent statistical significance (*p* < 0.05).

### Beta-diversity – variations in community composition driven by activity probe dependent microbe selection

**Fig. 4:**
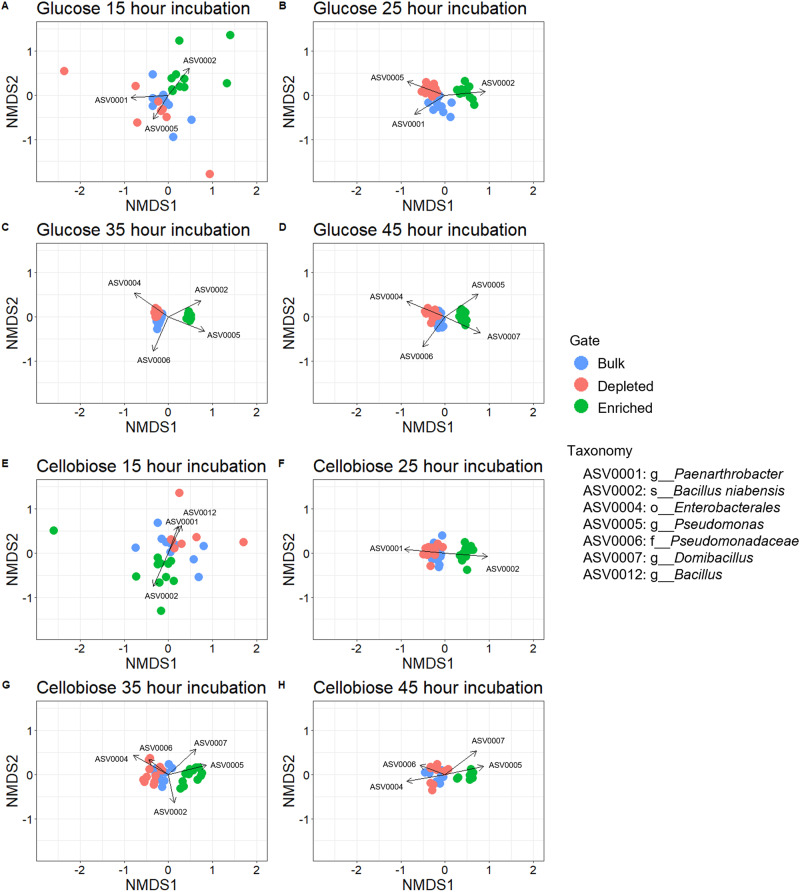
Ordination for GH4a probe. Nonmetric multidimensional scaling (NMDS) for Bray Curtis Dissimilarity of community relative abundance with SIMPER analysis with ASVs representing 70% of the sample differences for comparisons between enriched vs bulk and enriched vs depleted. **A**–**D** Sorted populations for glucose-MUB incubations. **A** Time point 1–15 h. **B** Time point 2–25 h. **C** Time point 3–35 h. **D** Time point 4–45 h. **E**–**H** Sorted populations for cellobiose-MUB incubations. **E** Time point 1–15 h. **F** Time point 2–25 h. **G** Time point 3–35 h. **H** Time point 4–45 h.

**Fig. 5:**
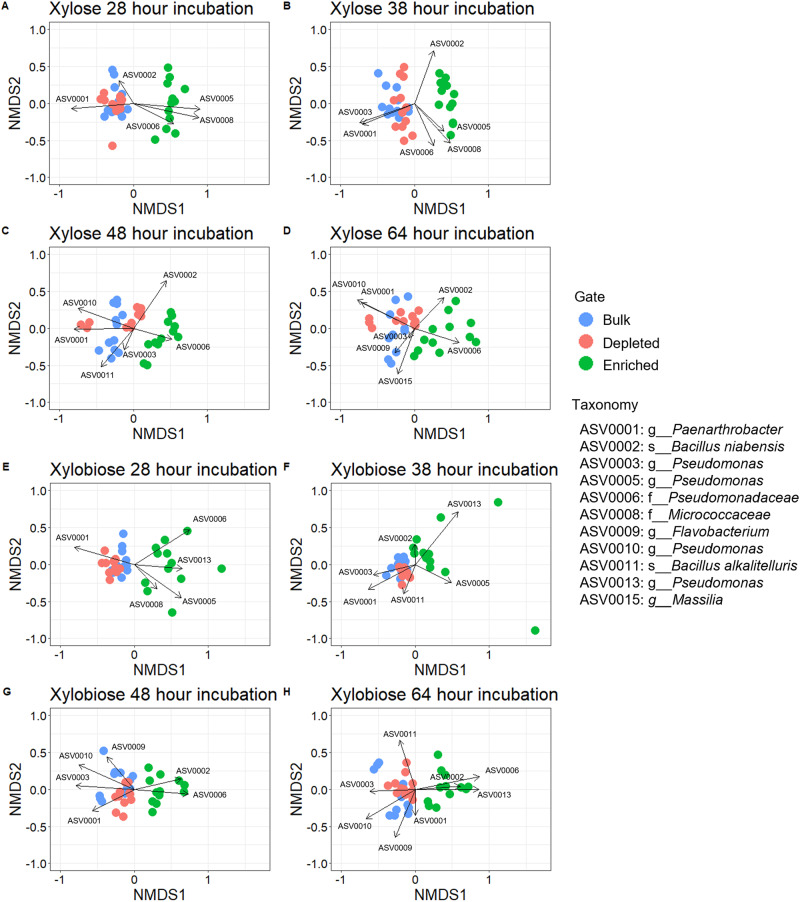
Ordination for SYF161 probe. Nonmetric multidimensional scaling (NMDS) for Bray Curtis Dissimilarity of community relative abundance with SIMPER analysis with ASVs representing 70% of the sample differences for comparisons between enriched vs bulk and enriched vs depleted. **A**–**D** Sorted populations for xylose-MUB incubations. **A** Time point 1–28 h. **B** Time point 2–38 h. **C** Time point 3–48 h. **D** Time point 4–64 h. **E**–**H** Sorted populations for xylobiose-MUB incubations. **E** Time point 1–28 h. **F** Time point 2–38 h. **G** Time point 3–48 h. **H** Time point 4–64 h.

Based on the discrete kinetic data and Shannon diversity, we hypothesized that the GH4a-ABP for cellobiosidase activity selected for a unique, enriched community composition distinct from the bulk and depleted communities. In addition to overall community composition, we wanted to identify the specific amplicon sequence variants (ASVs) that were driving the differences among sample types, which we hypothesize are the microbes possessing the function of interest. Intriguingly, the difference of community composition was significant for enriched samples compared to bulk or depleted samples for all time points incubated with glucose-MUB or cellobiose-MUB (*p* < 0.05; PERMANOVA, *p* value adjusted with Holm correction, Supplementary Table 1). To follow up the community composition, we investigated the contribution to the Bray Curtis dissimilarity for the ASVs composing the top 70% of the differences among the sample types at each time point using nonmetric multidimensional scaling (NMDS; Figs. 4 and 5). For both glucose-MUB and cellobiose-MUB, several ASVs covered the top 70% of difference at the first two time points, with a shift in ASVs being implicated for the dissimilarity at the latter two time points. *Bacillus niabensis* (ASV0002) was more abundant in enriched samples, whereas a member of *Paenarthrobacter* (ASV0001) and *Pseudomonas* (ASV0005) genera were the selective pressure and thus more abundant in bulk and depleted samples for the early time points. The shift that occurred for later time points implicated ASV0002 and ASV0005 for time point three and ASV0005 and *Domibacillus* (ASV0007) for time point four in the enriched samples. In bulk and depleted samples at time points three and four, *Enterobacterales* (ASV0004) and *Pseudomonadaceae* (ASV0006) were the cause of the differences from enriched samples. Interestingly, this trend in ASVs driving the differences was seen for both glucose- and cellobiose-MUB incubated samples with similar ASVs over the four time for the two MUBstrates (Fig. 4).

Despite lesser variation in kinetic data and higher initial α-diversity observed for xylose- and xylobiose-MUB incubated samples, the community compositions between enriched populations and bulk or depleted samples were significant at all four time points (*p* < 0.05; PERMANOVA, *p* value adjusted with Holm correction, Supplementary Table 1). In contrast to glucose- and cellobiose-MUB samples, xylose- and xylobiose-MUB samples composed five to eight ASVs making up the top 70% of differences among the samples (Fig. 5). Enriched samples were observed to be influences heavily by *Bacillus niabensis, Pseudomonadaceae*, and *Micrococcaceae* (ASVs 0002, 0006 and 0008) with multiple *Pseudomonas* ASVs (ASVs 0005 and 00013) while *Paenarthrobacter, Pseudomonas, Flavobacterium, Pseudomonas*, and *Bacillus alkalitelluris* (ASVs 0001, 0003, 0009, 0010, and 0011) were the top ASVs present in bulk or depleted samples.

## Discussion

In this study we used ABP-dependent microbial selection to enrich functionally distinct populations from a complex soil bacterial community and determine temporal kinetic enzyme activity. We successfully interrogated the soil community using two independent ABPs and subsequent four methylumbelliferone-tagged substrates to assess enzyme functions related to a lignocellulose-degrading phenotype.

By coupling activity-based probes and fluorescence-activated cell sorting (ABP-FACS) with live microbial communities we characterized and compared the probe labeled, unlabeled, and bulk microbial populations following cell sorting. The resultant kinetic assays displayed a prominent increase in activity for the enriched populations of glucose, cellobiose, and xylose incubations (Fig. 2). However, after the delayed response in activity was recorded for bulk and depleted populations, these samples reached similar concentrations of assay saturation at later time points. Metabolic phenotypic heterogeneity within the community could provide variation in specific microbes that were actively expressing the function of interest at the time of probing, while retaining this potential phenotype once the expressing microbes were separated via FACS. It has previously been shown that not all members of a microbial population are always active in soil [34, 35]. Rates of activity may vary depending on soil depth profile or functional redundancy [36, 37]. Microbes not expressing the function of interest for our probes would have remained in the depleted populations during FACS. The genomic potential to perform the activity could carry over for the depleted samples into the kinetic activity, hence why similar levels of substrate were consumed but requiring a longer incubation. Additionally, microbes performing the phenotype with extracellular enzymes in the original probed sample would be missed during FACS due to the lack of internalized fluorescent probe. Applying probes for protein enrichment to portions of the pre-sorted community coupled with proteomics could assist in answering if critical enzyme targets were missed for the FACS separation phenotype expressing microbes.

Functionally enriched microbial populations were distinguishable by kinetic assay. To reveal the microbes performing the functions of interest, we applied 16S rRNA gene amplicon sequencing to assign taxonomic identification of the ABP-sorted populations. Selective pressures were introduced with our cell extraction and incubation steps that decreased the initial microbial diversity prior to ABP application that may have hampered the quantity of probe targets. For GH4a-ABP-sorted samples, initial Shannon Diversity was significantly lower than the bulk or depleted samples suggesting less diversity of the probe-labeled microbes. Also seen in other systems, microbes active in the presence of complex polysaccharides (e.g., cellulose) were less diverse than total cell populationss suggesting a subset of the overall community are responsible for performing a cellulolytic phenotype [38, 39]. The increase in diversity of enriched populations at later time points could be indicative of extremely low abundance microbial community members beginning to respond more favorably to the culture condition post-sorting and causing the evenness value to increase. Alternatively, the enriched populations sorted via the SYF161 probe, targeting xylosidase and xylobiosidase activity, were more diverse at the earlier time point and maintained a consistent range of diversity over the course of incubation. Although both incubations started with the same soil inoculum, the diversity in microbial targets for the probes performed differently and selected for different microbes from the soil community. Specificity in ABP-targeting helps to elucidate community members responsible for the unique functions each of the probes were designed to label.

To further describe the trends seen in Shannon Diversity, the taxonomic identities via 16S rRNA gene sequencing were applied to the microbial populations to discern the microbes that were driving the differences in community composition. In the GH4a-ABP probed samples targeting glucosidase and cellobiosidase activity, there were seven ASVs driving most of the community differences across the sample time at the different time points. ASV0002, attributed to *Bacillus niabensis* was a major contributor for the differences in the enriched samples. *B. niabensis* was originally isolated from cotton waste compost [40] and subsequently shown to have β-glucosidase activity [41] further validating the phenotype specificity of the GH4a probe used in this study. During incubation for SYF161 sorted samples, a total of 11 unique ASVs were determined to cause most of the differences for the different sorted samples, slightly higher than for GH4a-ABP samples. These differences for both probe types were consistent throughout the incubation time and suggest distinct sorted communities capable of cellulose or xylose metabolism potentially indicative of further functional redundancy in the original soil community depending on the carbon source available. The microbial composition through relative abundance of the 16S rRNA genes varied over time showing that the sorted communities are not stable enriched consortia (Supplementary Fig. 5). To apply our approach for the generation of stable consortia, further work is needed to understand optimal growth conditions for the enriched members that dominate the communities at early time points.

ABP-enabled functional enrichment is preserved after sample incubation post-sorting as evident from the kinetic assay performed on the sorted populations. While we expected to identify microbes with a particular phenotype, continued activity towards that substrate of the depleted community opens future multi-omic experiments to test for genomic and expressed functionality of enriched versus depleted communities to better describe how the different communities function.

Successful expansion of our ABP-dependent approach to recover viable microbes for downstream kinetic assays demonstrates a powerful approach to query complex communities for desired phenotypes. The approach outlined here is a critical step toward high throughput identification of phenotypes in complex samples. Work to pair this approach with proteomics or metabolomics for an encompassing view of the activity of environmental microbes is in progress and will expand modeling efforts to further describe the phenotype interaction network of microbes in a complex sample. Due to the breadth of existing ABPP work, numerous ABPs have been reported for a wide variety of functions [42]. Combining this library of ABPs with our microbial phenotype selection pipeline will enable examination of several phenotypes across environments. This can include carbon cycling or plant growth promoting microbial phenotypes in soil, nutrient cycling or protective effects of host-associated communities, or built communities of microbes carrying out economically important biosynthetic functions. In addition, the advantage of our approach is both in identifying microbes that express a certain phenotype and in collecting these same microbes so that they remain viable, which will allow us to carry out subsequent multi-omic analysis of microbial communities enriched for desired functions. We also believe this approach can be further adapted to study microbial phenomics *in situ*, offering a way to identify microbial species that express a certain function but are not yet available as isolates in laboratory settings. The application of our approach has the potential to enhance synthetic biology and bioengineering applications and to discover active microbes and functional shifts in response to environmental or interspecies interaction cues.

### Supplementary information

Supplementary Information

Amplicon metadata table

Amplicon metadata table

Amplicon metadata table

ISMEJCOMMS-23-00084A-s11

## Data Availability

All sequence data associated with this study have been deposited in Datahub at PNNL (10.25584/1959236). The Qiime2 processed count data, taxonomy table, sample metadata and R code used for analysis are available as Supplementary Online Information.
