# Supplementary material for: Selection and enrichment of microbial species with an increased lignocellulolytic phenotype from a native soil microbiome by activity-based probing
Source: ISME Commun. 2023 Sep 30;3:106. doi: 10.1038/s43705-023-00305-w (PMC10542759; doi:10.1038/s43705-023-00305-w)
Supplement: Supplementary file 1 — Supplementary Information [file 43705_2023_305_MOESM1_ESM.pdf]

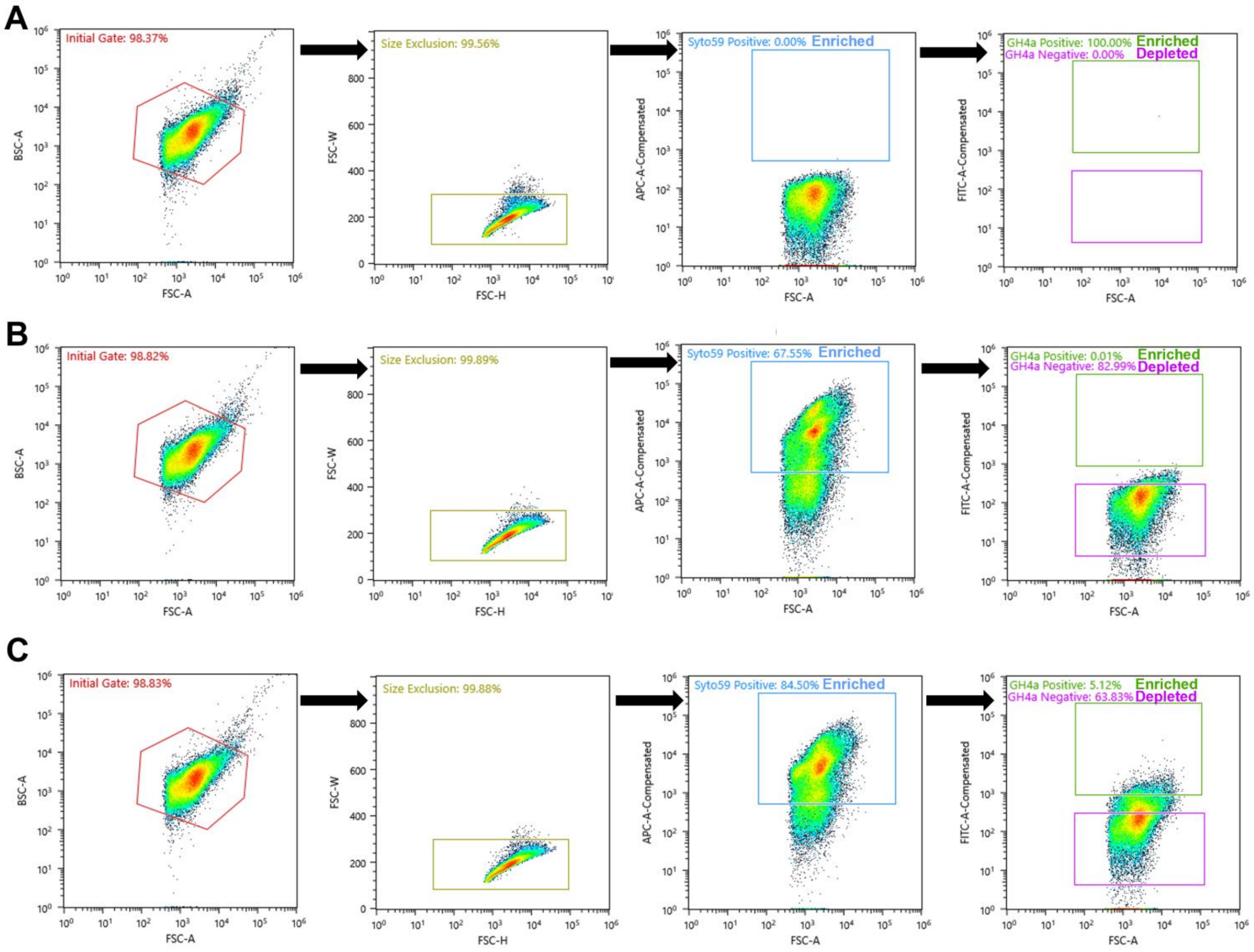

SI Figure 1. Sony SH800 sorter plots for sorting with GH4a activity-based probe. **(A)** Original set of sorter gates determined with an unlabeled control sample. **(B)** Sorter gates to determine biological events only containing the Syto59 signal. **(C)** Final sorter gates used to sort biological events containing signal from GH4a-ABP. Black arrows direction of nested plots.

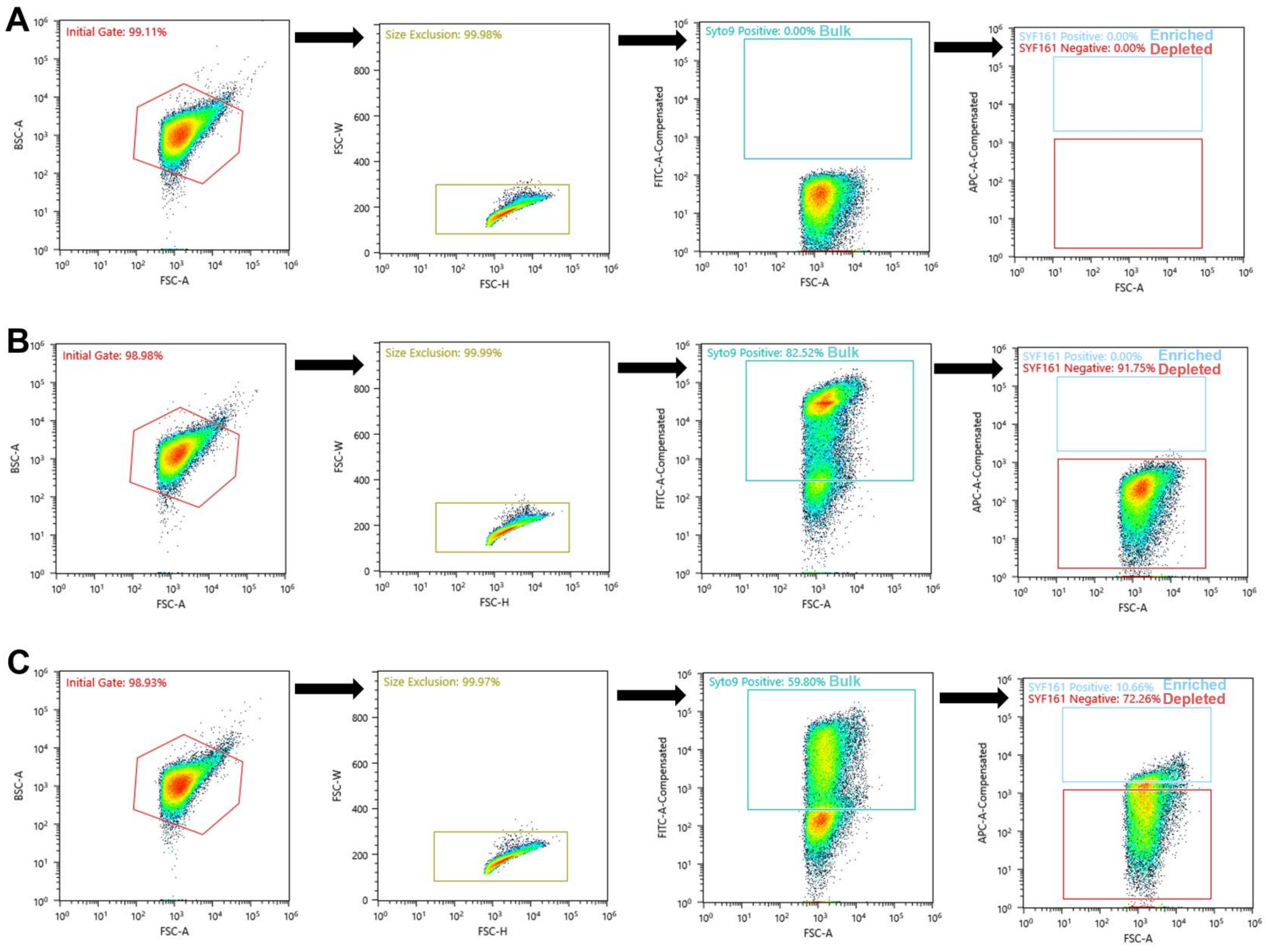

SI Figure 2. Sony SH800 sorter plots for sorting with SYF161 activity-based probe. **(A)** Original set of sorter gates determined with an unlabeled control sample. **(B)** Sorter gates to determine biological events only containing the Syto9 signal. **(C)** Final sorter gates used to sort biological events containing signal from SYF161-ABP. Black arrows direction of nested plots.

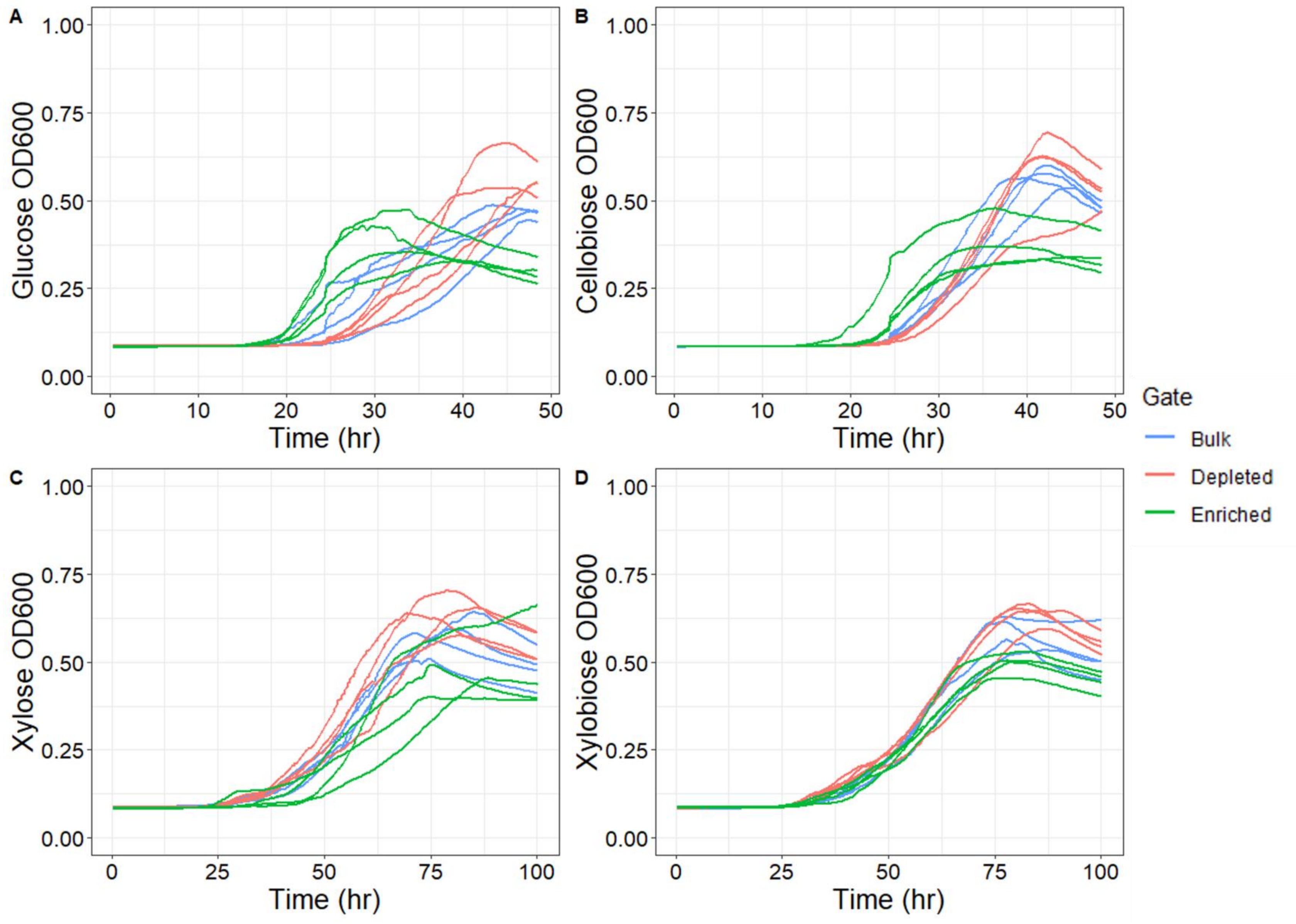

SI Figure 3. Optical density plots for the continuous plate reader assay (OD600). **(A)** GH4a sorted populations with glucose-MUB. **(B)** GH4a sorted populations with cellobiose-MUB. **(C)** SYF161 sorted populations with xylose-MUB. **(D)** SYF161 sorted populations with xylobiose-MUB.

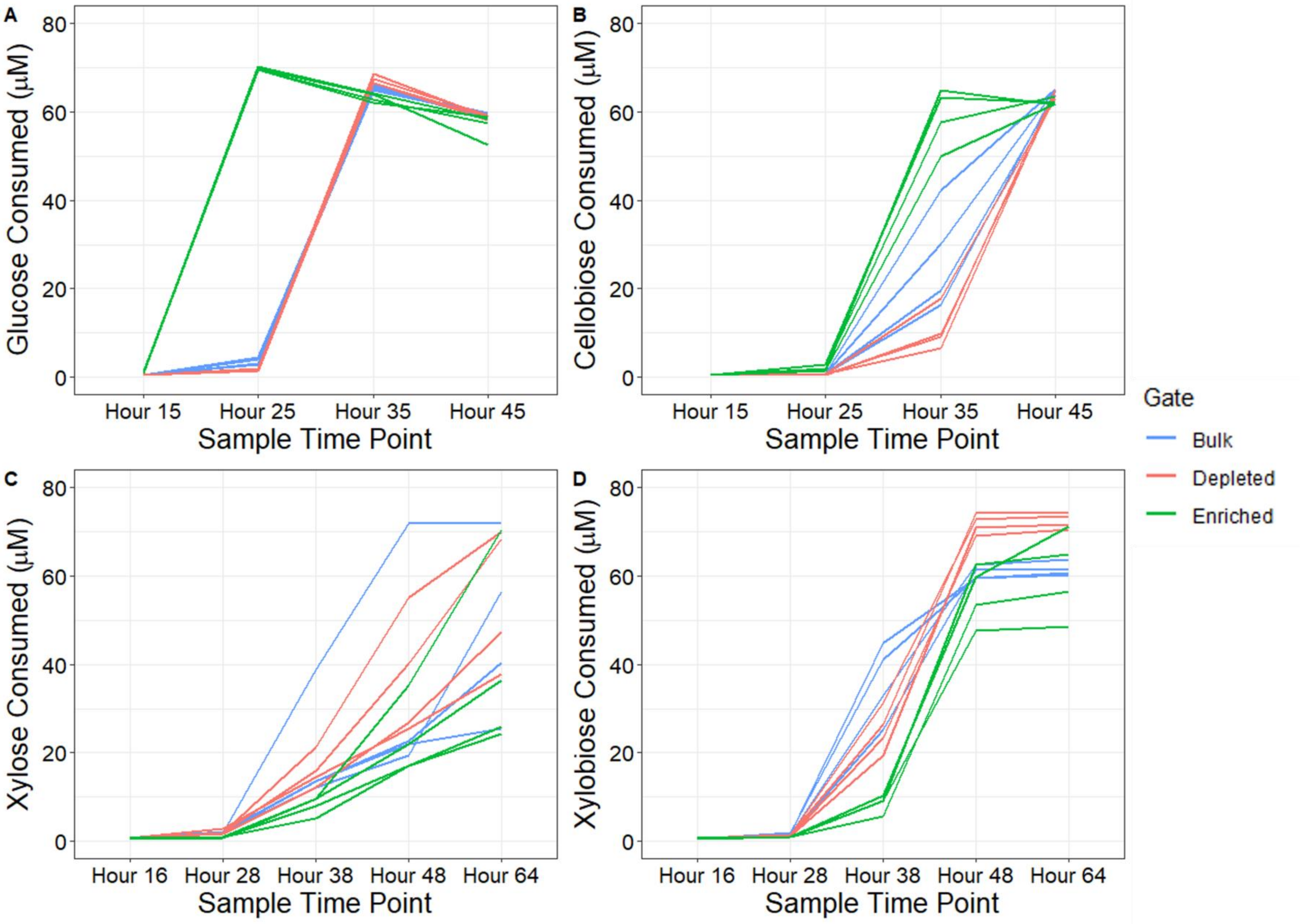

SI Figure 4. Discrete kinetic plots for substrate consumed (µM) at distinct time points. **(A)** Glucose-MUB incubated samples that were sampled at 15, 25, 35, and 45-hours during the incubation. **(B)** Cellobiose-MUB incubated samples that were sampled at 15, 25, 35, and 45-hours during the incubation. **(C)** Xylose-MUB incubated samples that were sampled at 16, 28, 38, 48, and 64-hours during the incubation. **(D)** Xylobiose-MUB incubated samples that were sampled at 16, 28, 38, 48, and 64-hours during the incubation.

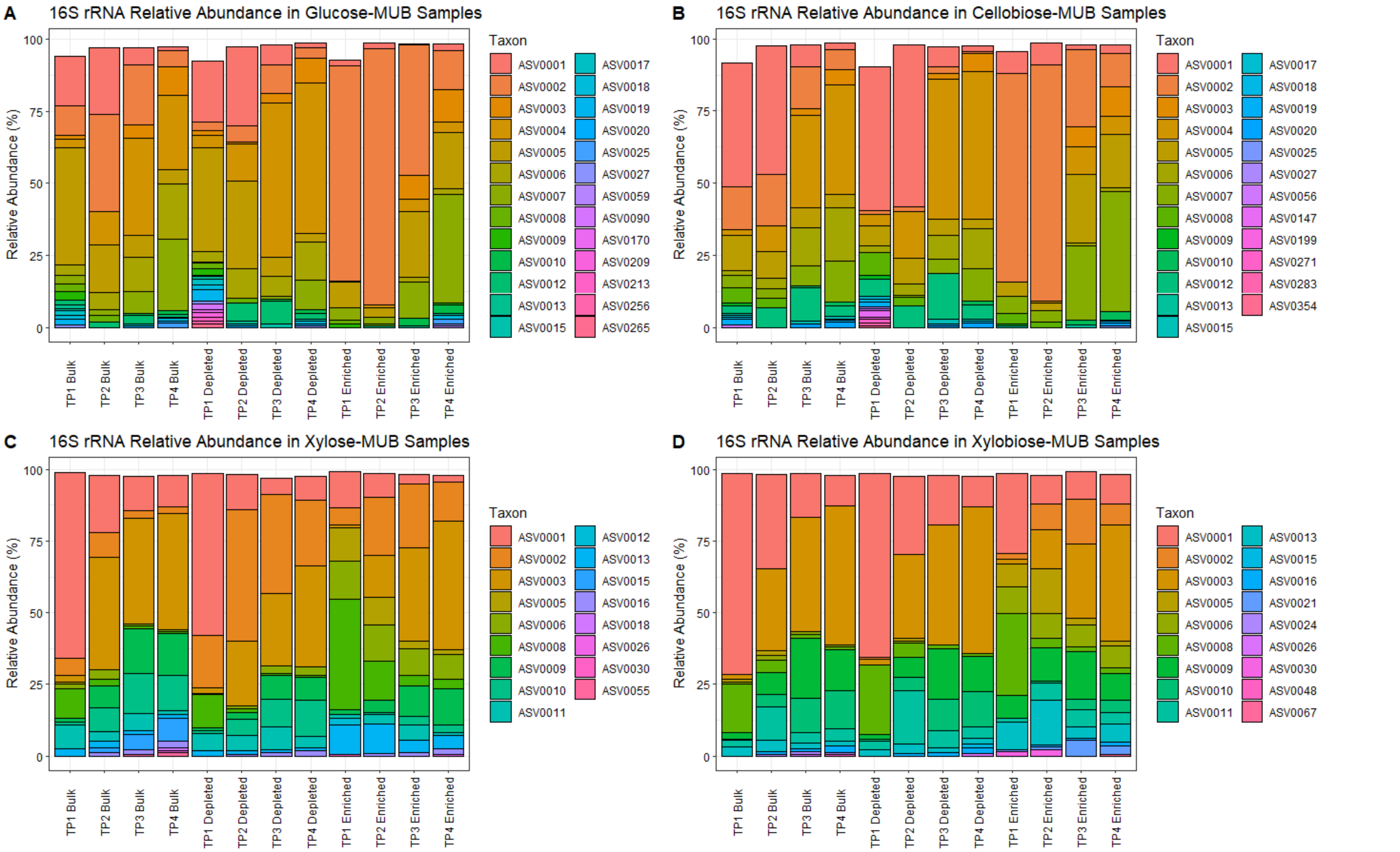

SI Figure 5. 16S rRNA gene relative abundances for averaged sorted incubations. Incubations from three sorter populations samples at four time points. Taxa represented as amplicon sequence variant (ASV) for taxa above 0.5 % relative abundance in the averaged samples. **(A)** Glucose-MUB incubated samples sampled at 15, 25, 35, and 45-hours during the incubation. **(B)** Cellobiose-MUB incubated samples that were sampled at 15, 25, 35, and 45-hours during the incubation. **(C)** Xylose-MUB incubated samples that were sampled at 16, 28, 38, 48, and 64-hours during the incubation. **(D)** Xylobiose-MUB incubated samples that were sampled at 16, 28, 38, 48, and 64-hours during the incubation.

Supplementary Table 1. PERMANOVA analysis of relative abundance community composition for FACS incubated samples. Pairwise comparison between sample types were compared by PERMANOVA using Bray Curtis dissimilarity with Holm p-value adjustment.

Time point	Sample comparison	Glucose			Cellobiose			Xylose			Xylobiose		
		F Model	R2	Adjusted p value	F Model	R2	Adjusted p value	F Model	R2	Adjusted p value	F Model	R2	Adjust[illegible] p valu[illegible]
TP1	Bulk v Depleted	1.57	0.0892	0.063	2.93	0.164	0.011	3.92	0.151	0.046	6.93	0.240	0.003
	Bulk v Enriched	72.48	0.801	0.003	41.96	0.688	0.003	71.07	0.764	0.003	90.69	0.805	0.003
	Enriched v Depleted	36.60	0.696	0.003	52.99	0.768	0.003	48.18	0.687	0.003	57.28	0.723	0.003
TP2	Bulk v Depleted	28.65	0.566	0.003	18.21	0.453	0.003	13.32	0.377	0.006	2.48	0.101	0.075
	Bulk v Enriched	97.46	0.816	0.003	278.69	0.927	0.003	14.62	0.399	0.003	21.43	0.493	0.003
	Enriched v Depleted	817.79	0.974	0.003	456.05	0.954	0.003	8.32	0.274	0.006	24.96	0.531	0.003
TP3	Bulk v Depleted	25.70	0.539	0.003	8.91	0.288	0.003	17.30	0.440	0.003	2.95	0.118	0.043
	Bulk v Enriched	59.95	0.732	0.003	18.86	0.462	0.003	13.87	0.387	0.003	9.54	0.303	0.003
	Enriched v Depleted	159.68	0.879	0.003	36.18	0.622	0.003	4.95	0.184	0.019	10.25	0.318	0.003
TP4	Bulk v Depleted	25.82	0.540	0.003	3.24	0.134	0.03	8.79	0.305	0.006	0.85	0.037	0.468
	Bulk v Enriched	74.09	0.771	0.003	30.51	0.629	0.003	9.25	0.316	0.003	12.57	0.364	0.003
	Enriched v Depleted	166.07	0.883	0.003	38.57	0.694	0.003	6.01	0.215	0.008	13.57	0.381	0.003
